# Biotic habitats as refugia under ocean acidification

**DOI:** 10.1093/conphys/coab077

**Published:** 2021-09-16

**Authors:** Laura J Falkenberg, Elliot Scanes, James Ducker, Pauline M Ross

**Affiliations:** 1 Simon F.S. Li Marine Science Laboratory, School of Life Sciences, The Chinese University of Hong Kong, Hong Kong SAR; 2 School of Life and Environmental Sciences, The University of Sydney, Camperdown, New South Wales, 2006, Australia; 3 Climate Change Cluster, University of Technology Sydney, Ultimo, New South Wales, 2007, Australia

## Abstract

Habitat-forming organisms have an important role in ameliorating stressful conditions and may be of particular relevance under a changing climate. Increasing CO_2_ emissions are driving a range of environmental changes, and one of the key concerns is the rapid acceleration of ocean acidification and associated reduction in pH. Such changes in seawater chemistry are anticipated to have direct negative effects on calcifying organisms, which could, in turn, have negative ecological, economic and human health impacts. However, these calcifying organisms do not exist in isolation, but rather are part of complex ecosystems. Here, we use a qualitative narrative synthesis framework to explore (i) how habitat-forming organisms can act to restrict environmental stress, both now and in the future; (ii) the ways their capacity to do so is modified by local context; and (iii) their potential to buffer the effects of future change through physiological processes and how this can be influenced by management adopted. Specifically, we highlight examples that consider the ability of macroalgae and seagrasses to alter water carbonate chemistry, influence resident organisms under current conditions and their capacity to do so under future conditions, while also recognizing the potential role of other habitats such as adjacent mangroves and saltmarshes. Importantly, we note that the outcome of interactions between these functional groups will be context dependent, influenced by the local abiotic and biotic characteristics. This dependence provides local managers with opportunities to create conditions that enhance the likelihood of successful amelioration. Where individuals and populations are managed effectively, habitat formers could provide local refugia for resident organisms of ecological and economic importance under an acidifying ocean.

## Teaser

Habitat-forming organisms, such as macroalgae and seagrasses, could ameliorate the effects of ocean acidification by countering the alterations to water chemistry through photosynthesis. Importantly, whether they will create refugia will be context dependent and influenced by local traits, meaning management at the local scale could fundamentally shape global change impacts.

## Introduction

Habitat-forming organisms can facilitate the occurrence and persistence of other organisms by creating favourable environmental conditions (Bulleri *et al.*, 2018). Such positive interactions influence species’ distributions and abundances, allowing organisms to occur in otherwise unsuitable areas (Bertness and Callaway, 1994; He *et al.*, 2013). The importance of habitat-forming organisms in ameliorating stress will increase as conditions continue to be altered under global climate change. That is, habitat-forming organisms may take on the role of creating climate change refugia, which can be thought of as areas where localized environmental conditions protect species from unfavourable or harmful conditions associated with broad changes to the Earth’s climate (Keppel *et al.*, 2012; Morelli *et al.*, 2016; Kapsenberg and Cyronak, 2019). Therefore, harnessing such positive interactions at local scales has the potential to limit the impacts of global climate change.

Increased CO_2_ emissions are dramatically altering the global carbon cycle and are set to continue to impact oceanic biota and ecosystems in complex ways from enhancing ocean acidification, through to increasing temperatures, modifying salinity, altering exposure to extreme events and more (IPCC, 2014). Around one-third to one-half of CO_2_ emissions are taken up by the Earth’s oceans (Feely *et al.*, 2004), where they alter carbonate chemistry and drive ocean acidification (Caldeira and Wickett, 2003; Feely *et al.*, 2004; Le Quéré *et al.*, 2015). Specifically, ocean acidification is associated with decreased pH, decreased concentration of carbonate ions and the reduced saturation state of calcium carbonate and mineral forms (i.e. calcite and aragonite) (Caldeira and Wickett, 2003). These chemical changes associated with ocean acidification can have particularly strong negative effects on calcifying species such as corals, coralline algae and shell-forming molluscs (Kroeker *et al.*, 2010; Ross *et al.*, 2011; Parker *et al.*, 2013); although effects on non-calcifiers are also recognized (e.g. non-calcifying macroalgae, sponges, plankton, invertebrates, fish; Heuer and Grosell, 2014; Nagelkerken and Connell, 2015). Anticipated effects on organisms, including both calcifying and non-calcifying species, include shifted energy budgets as more energy is required to prevent dissolution or maintain calcification, altered neurological functions and modified behaviour—all of which can affect organism growth, reproduction and survivorship (Pörtner and Farrell, 2008; Parker *et al.*, 2013; Gattuso *et al.*, 2015; Fernández *et al.*, 2019; Ducker and Falkenberg, 2020).

Negative effects of ocean acidification on calcifying organisms will have important ecological, economic and human health impacts (Falkenberg and Tubb, 2017; Falkenberg *et al.*, 2020). These impacts are anticipated as calcifying organisms, such as oysters, provide critical services along our coastlines including improved water quality, protection of shores and promotion of organic matter recycling (Lemasson *et al.*, 2017). Moreover, calcifying organisms are economically important due to their role in fisheries. That is, worldwide production of shelled mollusc calcifiers, which include oysters, cockles, clams, scallops, abalone and mytilids, recently reached ~17.3 million tonnes, representing 56.2% of the production of marine and coastal aquaculture (FAO, 2020). Where the occurrence of calcifying organisms is altered, this will modify the ability of humans to gain associated ecosystem services and affect our physical and mental health and well-being (Falkenberg *et al.*, 2020). Consequently, there is a need to develop management approaches that protect sensitive calcifying species, ecosystems, fisheries and aquaculture setups to help maintain the valuable marine services our societies depend upon (Mcleod *et al.*, 2013; Billé *et al.*, 2013; Falkenberg and Tubb, 2017). Where these systems are managed appropriately human societies could even benefit in the future; the management of habitat-forming organisms may allow for more efficient growth of calcifiers, meeting the needs of a growing population that is increasingly dependent upon marine resources.

The effects of global change on sensitive species and ecosystems will, however, be influenced by natural heterogeneity and the local context within which global change manifests. Where habitat-forming species are present, they have been shown to buffer against a range of stressful conditions. For example, shallow subtidal algal canopies are able to attenuate light stress (Meysman *et al.*, 2006; Bennett *et al.*, 2015). Similarly, in the intertidal zone, canopies reduce heat and desiccation stress during emersion (Leonard, 2000; Silliman *et al.*, 2011). Consequently, it has been suggested that biotic habitats could provide spatial refugia from the chemical stresses of ocean acidification (Unsworth *et al.*, 2012; Hendriks *et al.* 2014; Hurd, 2015; Greiner *et al.*, 2018; Groner *et al.*, 2018; Ricart *et al.*, 2021). These refugia would form where biotic habitats create areas within a species’ biogeographic range that experience less severe exposure to ocean acidification relative to other areas (Kapsenberg and Cyronak, 2019). Conditions can be altered in dense habitats as they are characterized by intense physiological processes and capacity to modify water chemistry of the surrounding environment (Dayton, 1985; Hofmann *et al.*, 2011; Cornwall *et al.*, 2013; Hurd, 2015). For example, a large-scale study considering 1000 km of the west coast of the USA over 6 years demonstrated that seagrass meadows can, indeed, alleviate low pH conditions for extended periods of time with seagrass-centred ecosystems exhibiting higher average pH (mean pH of 7.98 ± 0.002 SE) in comparison to non-vegetated areas (7.91 ± 0.001) (Ricart *et al.*, 2021). Similarly, the photosynthetic activity and respiration of seagrass beds have resulted in diel pH fluctuations of between 0.5 and 0.7 pH units (Unsworth *et al.*, 2012, and references therein) and 0.06 and 0.24 pH units (*Posidonia oceanica*; Hendriks *et al.*, 2014), with kelp beds found to drive diel fluctuations of 0.94 pH units (*Macrocystis pyrifera*; Cornwall *et al.*, 2013). These changes in water chemistry can then affect resident calcifiers by either reducing exposure to harmful conditions or enhancing adaptive capacity of the threatened species. The buffering capacity of photosynthesizers has been shown to benefit calcifiers in simulated ocean acidification experiments; however, these benefits are restricted to the immediate micro-environment surrounding the algae and seagrasses (Wahl *et al.*, 2018; Doo *et al.*, 2020).

Of particular interest within this context are photosynthetic organisms, specifically macroalgae and seagrasses, which can play similar ecological roles within ecosystems. Macroalgae can form ecosystem components ranging from the large, complex macroalgal canopies to low-lying coralline crusts (Hepburn *et al.*, 2011) in locations ranging from estuarine to deep subtidal locations (Layton *et al.*, 2020). This diversity is reflected in the three evolutionary distinct clades of macroalgae: Rhodophyta (red algae), Chlorophyta (green algae) and Phaeophyceae (brown algae). However, these algae can also be placed into ecophysiologically defined groups based on their physiological functioning—particularly photosynthetic mechanisms—which will likely lead to a diversity of responses to future ocean acidification (discussed further in Macroalgae and seagrasses as stress alleviators and refuge providers). Seagrasses form meadows in nearshore waters, commonly estuarine and wind-swept intertidal areas (Duarte *et al.*, 2008, and references therein). This group of plants consists of around 60 similarly functioning angiosperm species within two families (Potamogetonaceae and Hydrocharitaceae) encompassing 12 genera. Consequently, seagrasses are relatively similar in terms of their physiological processes, including photosynthesis, and so may have similar potential to form refugia under future climate conditions (also discussed further in Macroalgae and seagrasses  as stress alleviators and refuge providers). While we recognize the importance of habitat formers and the refugia they provide under contemporary stress, whether these benefits will be maintained at wide spatial scales under future conditions remains unclear. This uncertainty results as future abiotic conditions have the potential to modify the persistence of the habitat former itself, as well as the influence it has on the surrounding environment. Here, we will provide a qualitative narrative synthesis (*sensu*  Falkenberg *et al.*, 2018) regarding the potential for key habitat-forming organisms, specifically macroalgae and seagrasses, to provide refugia under future ocean acidification. Under this narrative synthesis framework we will also emphasize the local context dependency. Finally, we will outline the resultant management approaches that could be implemented to promote the continued ecological, economic and human health benefits we currently enjoy from calcifying marine organisms.

**Figure 1:**
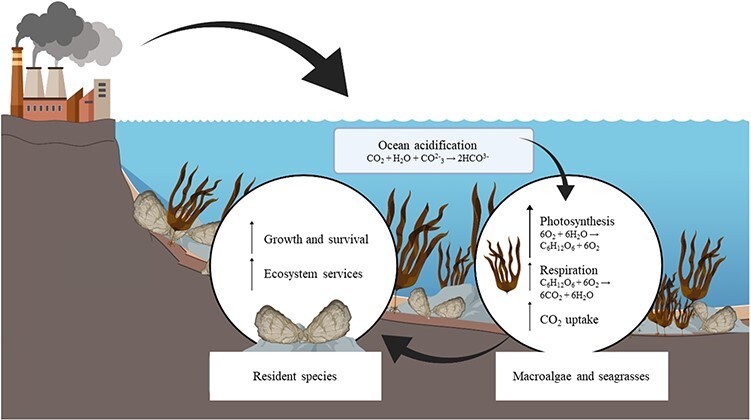
Habitat-forming species, such as macroalgae and seagrasses, can form refugia for resident species, including calcifying organisms. As human activities add CO_2_ to the atmosphere, it will be taken up by the ocean and lead to acidification, increasing the importance of algal photosynthesis and CO_2_ uptake as this can reduce biotic effects of acidification on calcifying organisms, increasing their growth and survival, as well as the benefits we receive from associated ecosystem services.

## Occurrence of habitat-associated refugia in future acidified oceans

Identifying whether habitat-forming species will provide refugia under climate change requires understanding if they will be able to persist and continue to provide benefits under future conditions. Specifically, to form effective refugia, habitat-forming species will need to be characterized by (i) an ability to function as stress alleviators, (ii) an ability to maintain viable populations and (iii) consistent morphology and phenology (Bulleri *et al.*, 2018). A diverse range of organisms have the potential to create habitat associated refugia including photosynthetic organisms such as macroalgae, seagrasses, mangroves and saltmarshes (Ross and Adam, 2013). Here we choose to focus on one of the most promising groups, the macroalgae and seagrasses, and consider if they will meet the conditions described above to provide benefit to resident organisms even under a future acidified ocean.

### Macroalgae and seagrasses as stress alleviators and refuge providers

Where primary producers form habitats, such as kelp forests and seagrass meadows, their metabolic activity has the potential to modify water chemistry of the surrounding environment to an extent that it may locally ameliorate long-term decreases in pH (Fig. 1). That is, primary producers such as macroalgae and seagrasses have a large capacity to fix CO_2_ through photosynthesis (Harrison and Hurd, 2001; Chung *et al.*, 2013; Pacella *et al.*, 2018). Consequently, habitats formed by these primary producers are characterized by periods of intense metabolic activity that modify the surrounding environment, particularly in coastal areas (Dayton, 1985; Hofmann *et al.*, 2011; Cornwall *et al.*, 2013; Hurd, 2015). During the day, rates of photosynthesis are increased, resulting in CO_2_ being removed from the seawater and increasing pH. In contrast, during the night, rates of respiration are increased, resulting in CO_2_ being released to the surrounding seawater and reducing pH (Hofmann *et al.*, 2011; Cornwall *et al.*, 2013). The magnitude of resulting change in pH will vary among locations and species considered; in kelp beds (*M. pyrifera*) this physiological activity can lead to pH ranging by ~1 pH unit, from 7.92 to 8.86 pH units, over a diel cycle (Cornwall *et al.*, 2013). Similarly, in seagrass systems, studies from a range of locations (the Philippines, Great Barrier Reef, Mediterranean) have highlighted diel changes in pH of between 0.5 and 0.7 pH units, with peak values reached around midday when photosynthesis is at its maximum (Unsworth *et al.*, 2012, and references therein). Such changes can also persist over longer periods, with seagrass-centred ecosystems exhibiting higher average pH (mean pH of 7.98 ± 0.002 SE) in comparison to non-vegetated areas (7.91 ± 0.001) (Ricart *et al.*, 2021).

The influence of primary producers on water chemistry likely influences the resident organisms we observe and their capacity to deal with anticipated change. For example, strong effects of primary producers on the diurnal variability of water chemistry results in the associated organisms experiencing more extreme conditions as well as more variability in conditions. In terms of more extreme conditions, the increased daytime pH in the presence of primary producers can create favourable conditions for shellfish calcification (Roleda *et al.*, 2015; Gobler *et al.*, 2017). Where global pH is reduced under ocean acidification, these daily increases may provide important opportunities for calcification. Moreover, photosynthetic organisms can produce higher average mean pH over longer periods (Ricart *et al.*, 2021). In the absence of primary producers, and the associated increases in pH, ocean acidification is widely anticipated to negatively affect calcification (Nagelkerken and Connell, 2015; Figuerola *et al*., 2021).

For refugia to be provided under ocean acidification, macroalgal and seagrass photosynthesis will need to continue modifying water chemistry. It is anticipated that the increased CO_2_ availability associated with ocean acidification will lead to increased rates of photosynthesis by relieving macroalgae and seagrasses of their current under-saturation of dissolved inorganic carbon (Koch *et al.*, 2013). The precise response observed will, however, depend on the photosynthetic mechanisms, and carbon species, used by the alga or seagrass of interest (e.g. Hepburn *et al.*, 2011; Falkenberg *et al.*, 2013b; Kim *et al.*, 2016). In terms of macroalgae, the majority have carbon concentrating mechanisms (CCMs) that are used in the active uptake of CO_2_ and/or bicarbonate (HCO_3_^−^) and elevation of concentrations at the site of carbon fixation, although some do use dissolved CO_2_ entering by diffusion (Beardall and Giordano, 2002). Consequently, algae with CCMs are generally expected to gain little benefit from enriched CO_2_ while, in contrast, algae that rely on diffusion are anticipated have increased photosynthetic assimilation and productivity (Kübler *et al.*, 1999; Hurd *et al.*, 2009; Falkenberg *et al.*, 2013b). It is worth noting, however, that some seaweeds with CCMs can benefit from ocean acidification as the energy saved from downregulated CCMs can be used for other physiological activities, such as nitrogen assimilation (Gao *et al.*, 2016; Xu *et al.*, 2017)*.* It has been estimated that >50% of red macroalgae and <20% of brown and green macroalgae are non-CCM (Hepburn *et al.*, 2011; Cornwall *et al.*, 2015). Therefore, we could expect that adults of the CCM species would continue to modify water conditions to a similar extent, while the non-CCM species could potentially have stronger influences on water chemistry, providing more effective refugia. Importantly, however, the morphology of these groups varies, influencing their capacity to be considered as habitat formers. That is, in a kelp forest community species exhibiting characteristics of a functional CCM were the large brown kelps and large kelp-like fucoid species, which characterize the canopy and sub-canopy guilds (Hepburn *et al.*, 2011) and are widely thought of as habitat forming (e.g. Smale *et al.*, 2013; Vásquez *et al.*, 2014). In contrast, the majority of non-CCM species were diminutive (<20 cm long) turf-forming rhodophytes that exhibited a range of morphologies (filamentous, crustose, terete, bladed), with these fleshy and filamentous red algae currently having limited ecological roles (Hepburn *et al.*, 2011). In terms of seagrasses, many are similar to algae in that they appear able to take up both CO_2_ and HCO_3_^−^, with concentrations likely enhanced by CCMs (Larkum *et al.*, 2017). Despite this capacity to use HCO_3_^−^, the majority of seagrasses currently appear carbon limited (reviewed in Koch *et al.*, 2013). Consequently, it is anticipated that increased CO_2_ availability could have positive effects on the photosynthesis of seagrasses (Short and Neckles, 1999; Fabricius *et al.*, 2011; Koch *et al.*, 2013) and, therefore, their capacity to form habitats (e.g. Hyndes *et al.*, 2003), alter water chemistry and provide refugia. Seagrasses are competitively dominant at highly acidified CO_2_ vent sites, indicating that near future (2100) CO_2_ concentrations will likely not exceed upper thresholds of CO_2_ tolerance in seagrasses (reviewed in Koch *et al.*, 2013).

### Macroalgal and seagrass population maintenance

For macroalgae and seagrasses to provide habitat refugia in the future, their populations will need to persist under modified conditions (including ocean acidification, as well as other abiotic and biotic traits that are discussed further in Other global change parameters) and be sustained by young individuals introduced via reproduction and continued growth of adults. In the context of ocean acidification specifically, experiments conducted to date considering the reproduction and growth of macroalgae and seagrasses under future conditions have found largely neutral, or positive, effects. That is, ocean acidification has led to no significant difference in the rate of zoospore germination in kelp (Olischläger *et al.*, 2012) and positive effects on reproductive output (including flowering frequency) in seagrasses (Palacios and Zimmerman, 2007). While there has been a reduction of germination success of giant kelp under ocean acidification, it is worth noting that this occurred at high *p*CO_2_ levels (~1800 μatm) not forecasted to occur in the near future (Gaitán-Espitia *et al.*, 2014). Similarly, mature algae exposed to elevated *p*CO_2_ conditions have found to demonstrate increased specific growth rates (Gao *et al.*, 2017). Consequently, it is anticipated that under ocean acidification, at least in the short term, macroalgal and seagrass reproduction and growth will be maintained, if not enhanced.

### Macroalgal and seagrass morphology and phenology

For habitats to provide refugia, the morphology and phenology of individuals will need to be sustained (Bulleri *et al.*, 2018). In the context of ocean acidification, there is evidence that macroalgal shape could be modified under ocean acidification. That is, *Fucus vesiculosus* individuals exposed to future ocean acidification levels have been found to increase their surface area over time (i.e. growth rate) to a greater extent than control individuals (Kinnby *et al.*, 2021), yet other studies have found that growth can be unchanged, or even lower, under high *p*CO_2_ (*F. vesiculosus*, Gutow *et al.*, 2014; Takolander *et al.*, 2019). For seagrasses there is evidence that their leaf size, as well as shoot and leaf density will be unaffected by ocean acidification (Palacios and Zimmerman, 2007; Guilini *et al.*, 2017), and a growing body of research highlights the potential for shoot density to be increased (Hall-Spencer *et al.*, 2008; Collier *et al.*, 2018; Mecca *et al.*, 2020). If seagrass shoot density were to remain unchanged, the effect of seagrass on water chemistry would also be maintained; however, it has been identified that there is a significant positive relationship between the area of leaf available for photosynthesis and the aragonite saturation state (Hendriks *et al.*, 2014).

## The context dependence of refuge provision by macroalgae and seagrasses

The influence of habitat-forming species, their capacity to provide refugia and the extent of resulting benefits will be context dependent and site specific, influenced by abiotic and biotic conditions of the surrounding environment. In terms of abiotic conditions, influential features include hydrodynamic regimes, which incorporate flow rates, vertical mixing and upwelling, input of freshwater and contaminants, depth, seasonality, as well as other global change parameters (e.g. warming, hypoxia). Similarly, biotic conditions can influence the occurrence of refugia, with conditions of particular relevance including which habitat-forming species are present and their physiological characteristics, the ratio of habitat-forming to resident organisms, as well as the presence of other interacting organisms and diseases.

### Abiotic conditions

#### Flow rates

Hydrodynamic regimes incorporate a range of parameters associated with water movement, many of which can influence the capacity of habitat formers to provide refugia. Of particular interest in this context are flow rates, as high flow rates can wash away, and thereby neutralize, the expected benefits of habitat formers (Mongin *et al.*, 2016). Consequently, refugia are less likely to form in high flow areas. It is worth noting, however, that the presence of habitat-forming species can modify the flow rate experienced, potentially facilitating the creation of refugia; that is, where habitat formers are introduced they can reduce current speeds, increase residence times and potentially accentuate the buffering effect (Hendriks *et al.*, 2014; Hurd, 2015; Fernández *et al.*, 2019). The reduction in flow rate can, however, have consequences for the habitat-forming species itself. For example, reduced speed can increase the diffusion boundary layer surrounding macroalgal blades and slow their nutrient uptake (Hurd, 2000), which influences growth rates. Such a reduction in nutrient uptake rates may be counteracted where resident organisms are present as the nutrient-rich waste they release would be retained (Dumbauld *et al.*, 2009; Ferriss *et al.*, 2016), increasing nutrient availability. However, the accumulation of waste on macrophytes could negatively affect their photosynthetic efficiency, particularly in the microscopic stages (Harrington *et al.*, 2005; Jiang *et al.*, 2015). Moreover, the accumulation of nutrients has the potential to enhance the expansion of other species, which include otherwise ephemeral algae that may provide altered ecosystem services compared to the previously dominant habitat-forming species (e.g. habitat complexity, food resources, pest control; Eriksson *et al.*, 2002; Kraufvelin *et al.*, 2006; Gorman *et al.*, 2009), particularly in sheltered coastal areas (Madsen *et al.*, 2001; Kotta *et al.*, 2009). Finally, low flow rates can lead to the accumulation of epibotia, which can create a range of complex effects including coverage of seagrass or algal blades that reduces their ability to photosynthesize and can lead to tissue degradation, alteration of water chemistry through photosynthesis and potential further modulation of ocean acidification and consumption and removal of epiphytes and effects on the persistence of associated seagrass meadows (e.g. by the sea hare *Phyllaplysi taylori*) (Bulthuis *et al*. 1986; Silberstein *et al.*, 1986; Hughes *et al.*, 2010).

#### Vertical mixing and upwelling

Vertical mixing associated with upwelling can influence the formation of ocean acidification refugia. Specifically, upwelling events can drive wide fluctuations in seawater chemistry with alternating periods of high CO_2_ and low pH, and low CO_2_ and high pH (Torres *et al.*, 2011). It is important to note that regions will be differentially affected by climate change-driven shifts in upwelling (Sydeman *et al.*, 2014); refugia established near contemporary upwelling areas may, therefore, show regionally specific sensitivities to future changes. While such variability can make it difficult to establish refugia, recognizing those areas characterized by periods of naturally reduced pH, which may experience exacerbated negative effects of ocean acidification (Cai *et al.*, 2011), will be key as intervention may be particularly important in such areas. Indeed, the negative effects of upwelling on calcifying organisms have already been observed in some regions. Oyster aquaculture along the US west coast was affected in 2006 when upwelling of CO_2_-rich waters drove production failures and impacted the associated industry. This experience has led to the introduction of a range of adaptive procedures to avoid the negative effects of incursion of CO_2_-rich waters. Two key changes that have been widely implemented are as follows: (i) during periods of upwelling, seawater is taken in during the afternoon at the diurnal low of CO_2_; and (ii) calcium chloride and sodium carbonate are added to the seawater to increase aragonite saturation levels. Additionally, one oyster operation has opened a hatchery in Hawaii, with larvae flown back at 1–2 weeks of age for rearing (as summarized in Branch *et al.*, 2013). Where photosynthetic organisms are present, they may similarly influence the local water chemistry, producing more favourable conditions for aquaculture.

#### Input of freshwater and contaminants

Water chemistry can be modified by the input of freshwater to a system, as observed in estuarine systems (Vargas *et al.*, 2016). Waters in these systems are typically characterized by low pH, carbonate ions and alkalinity (Duarte *et al.*, 2013; Waldbusser and Salisbury, 2014; Scanes *et al.*, 2020a). These conditions could, similarly to increased upwelling, exacerbate the negative effects of ocean acidification (Cai *et al.*, 2011). Consequently, establishing habitat-associated refugia in these areas could be particularly beneficial. That is, even short exposure to higher pH, as results diurnally in macroalgal or seagrass beds, might help calcifying organisms tolerate sustained low pH conditions in the surrounding environment (Frieder *et al.*, 2014; Enochs *et al.*, 2018). It is worth noting, however, that where runoff is particularly extensive, it may modify water chemistry and neutralize the benefits of habitat-associated refugia. Runoff can introduce contaminants to the water column, which modify the potential for habitat-forming refugia to occur. For example, the input of untreated sewage or agricultural runoff at local scales can be associated with nutrients and contaminants that then distribute toxic materials (Burridge *et al.*, 1996; Coleman *et al.*, 2008), enhance the occurrence of competitors (Connell *et al.*, 2008) and epiphytes (Silberstein *et al.*, 1986), as well as prompt high turbidity limiting macrophyte photosynthesis (Tait, 2019). For these reasons, habitat-associated refugia may be best suited to coastal regions with lower rainfall and lower riverine and estuary flow such as Australia (Roy *et al.*, 2001; Scanes *et al.*, 2020a), South Africa (Adams and Van Niekerk, 2020), the Mediterranean (Franco *et al.*, 2008) and western North America (Jacobs *et al.*, 2011). Within these regions, there are likely to be distinct effects of hydrodynamic regimes on macroalgae and seagrasses, with seagrasses largely found in estuarine and wind-swept intertidal areas, and macroalgae occurring in a species-dependent manner from estuarine to deep subtidal locations. Across all regions and habitat types it is likely that areas with lower watershed development, or good water treatment infrastructure, would experience the most limited transport of pollutants and, as such, be best for refugia.

#### Light availability

Light availability will be an important factor in determining where habitat refugia form given that this abiotic factor limits the occurrence of algae and seagrasses. That is, light attenuation, which is associated with depth (along with other factors), has been conservatively proposed to restrict productivity and the sites where macrophyte and seagrass habitats can be located to the first few meters of the water column (Beer *et al.*, 2016). For some notable brown algal species, such as the giant kelp *M. pyrifera*, the depth for effective suspended growth has been identified at around 3 m (this is the depth at which the maximum growth responses; greatest biomass was achieved in Varela *et al.*, 2018). However, red macroalgae have the accessory pigments phycoerythrin and phycocyanin that facilitate their growth and productivity at deeper locations (of below 4 m depth and up to ~13 m depth; Fernández, *et al.* 2019, and references therein) and research has indicated greater photosynthesis under ocean acidification in red algae species (e.g. *Neosiphonia harveyi*; Olischläger and Wiencke, 2013). In terms of seagrasses, depth has also been found to be a critical determinant of occurrence. In one particular study, long-term restoration success was restricted to a depth range of 0.5–0.8 m below mean sea level (Aoki *et al.*, 2020). It is important to note, however, that the role of depth will also be influenced by water clarity, which differs across latitudes and varies with local conditions (Luis *et al.*, 2019). Consequently, although there will be differences based on local conditions, the location of habitat refugia will likely be largely limited to regions with shallower depths.

#### Seasonality

Seasonality will have an influence on the abiotic conditions experienced in marine habitats and the ecological impacts of refugia. That is, a long-term study of seven seagrass meadows along the US west coast identified that maximal pH elevations occurred in spring and summer, during the seagrass growth season (Ricart *et al.*, 2021). The effect will also be determined by the occurrence of low-pH events, the seasonal timing and duration of which will vary regionally. In the context of the US west coast region considered above, there are two important oceanographic regions: Northern California, which is dominated by the California Current and is an upwelling region with cooler and more CO_2_-rich waters brought up near the surface seasonally; and Southern California, which is dominated by the Southern California Counter-current and is a region with less intense upwelling (Ricart *et al.*, 2021). Notably, in this region the magnitude and duration of pH amelioration events within seagrass meadows are larger than those of seasonal pH exacerbation events. That is, in upwelling areas, the low pH period often occurs in spring and summer, aligning with times when seagrass pH amelioration is also highest. The upwelling may actually enhance seagrass photosynthetic rates, and therefore amelioration at these times, by leading to an oceanographic influx of both aqueous CO_2_ and nutrients into the coastal zone (Ricart *et al.*, 2021, and references therein).

#### Other global change parameters

While we focus on the role of climate change through its effect on ocean acidification, we acknowledge that other abiotic conditions will also be modified in the future, with the potential that they too will influence the responses observed. For example, ocean warming could modulate the effect of ocean acidification, with some evidence that organisms are more sensitive where co-exposed to warming (Kroeker *et al.*, 2013; Nagelkerken and Connell, 2015). In addition to modifying the responses of biota, changes in temperature could also modify other abiotic traits; for example, temperature increases are a key reason why greater stratification is being observed across the global ocean (Li *et al.*, 2020) and temperature is known to have impacts of microbiomes (Scanes *et al.*, 2021), which could influence the formation of refugia through impacts on upwelling and vertical mixing, as well as health and survival. Similarly, there are also other numerous changes that will be observed in marine systems related to climate change (such as hypoxia, sea level rise, increased storm frequency, etc.; Harley *et al.*, 2006) and further complicate organism responses to ocean acidification. Moreover, non-climate change global impacts, such as deforestation or habitat destruction—through activities such as aquaculture, invertebrate harvesting, harbour activities or direct removal—will also play a direct role in determining the occurrence of refugia (Bustamante and Castilla, 1990; Nordlund and Gullström, 2013; Thomsen *et al.*, 2020).

### Biotic conditions

#### Which habitat-forming species are present and their physiological characteristics

The type of habitat-forming species present will influence the formation, and persistence, of refugia given their varied physiological characteristics. Of particular relevance when considering macroalgae and seagrasses are the photosynthetic traits, as this will influence their capacity to take up extra CO_2_ and reduce it in the water column (for example, species that use passive vs. active CO_2_ uptake mechanisms, which is discussed in more detail in Macroalgae and seagrasses  as stress alleviators and refuge providers, or the presence of accessory pigments, which is discussed in Light availability).

#### The ratio between habitat-forming and resident organisms

The extent of ocean acidification buffering will be dependent upon the efficiency of CO_2_ uptake which will, in turn, be determined by the ratio between habitat-forming species and the resident species. That is, in dense macroalgal or seagrass habitats, seawater pH can be increased to more favourable values for the calcification of associated organisms (e.g. 0.4 pH units higher than ambient during the day; Cornwall *et al.*, 2013). In turn, resident organisms can excrete nutrient-rich waste products and release CO_2_ via respiration, which can favour the growth of macroalgae (Chung *et al.*, 2013; Duarte *et al.*, 2017). However, the ratio of these functional groups could prevent the desired beneficial effects. For example, if the relative abundance of resident organisms is too high, this can lead to increased waste products and nutrients in the water column, smothering the macrophytes (Harrington *et al.*, 2005; Jiang *et al.*, 2015), and providing benefit to fast-growing competitors, which are able to replace the macrophytes (Eriksson *et al.*, 2002; Kraufvelin *et al.*, 2006; Gorman *et al.*, 2009).

#### The presence of other interacting organisms, such as herbivores

While we have focussed here on the habitat formers and calcifying organisms, they do not occur in isolation, but rather in complex ecosystems. The presence, or absence, of other key ecosystem components will have an influence on the occurrence of refugia. For example, overgrazing by herbivores, often sea urchins, has resulted in the decline of kelp forests (Filbee-Dexter and Scheibling, 2014; Ling *et al.*, 2015). Sea urchins have driven, and likely will continue to drive, such change where their predators (e.g. otters, fishes, lobsters) are removed from an ecosystem (Shurin *et al.*, 2010). Sea urchins are, however, also vulnerable to ocean acidification and are expected to experience less recruitment and development of juveniles in a future ocean (Byrne *et al.*, 2011; Foo *et al.*, 2012). It is important to note that grazers can also have positive effects on habitats. For example, the sea hare *P. taylori* removes epiphytes from seagrasses, influencing the persistence of this habitat former (Hughes *et al.*, 2010). Interactions between predators and their prey, and grazers and their food, have been found to be altered by ocean warming and acidification (Falkenberg *et al.*, 2013c; Russell *et al.*, 2013; Sampaio *et al.*, 2017; Wright *et al.*, 2018). Consequently, the occurrence of organisms at a range of trophic levels is an important component of the context dependency and efficacy of habitat refugia.

#### Disease

The distribution and abundance of species will be further influenced by diseases. A key example of a microbial agent prompting the decline of a submersed aquatic plant is the disease of eelgrass, *Zostera marina* L. (Shearer, 1994), which may also have affected its seed dispersal and connectivity (Tol *et al.*, 2017). This disease can interact with other stressors identified such that the effect is enhanced. For example, the removal of herbivores (such as the green turtle and dugong) has been linked to the proliferation of the wasting disease that has caused these significant declines of seagrass meadows (Jackson *et al.*, 2001). Conversely, the presence of Pacific oysters may be beneficial as they can filter out pathogens which cause this eelgrass wasting disease. Moreover, abiotic traits can influence the occurrence of disease, with climate change anticipated to increase pathogen development and survival rates, disease transmission and host susceptibility (Harvell *et al.*, 2002). The beneficial relationships between organisms may become even more important under such scenarios, with a recent experiment highlighting that when exposed to natural concentrations of the pathogen under high *p*CO_2_ conditions, eelgrass can benefit from co-culture with oysters (Groner *et al.*, 2018).

## Application of habitat formers in ocean acidification adaptation

**Figure 2:**
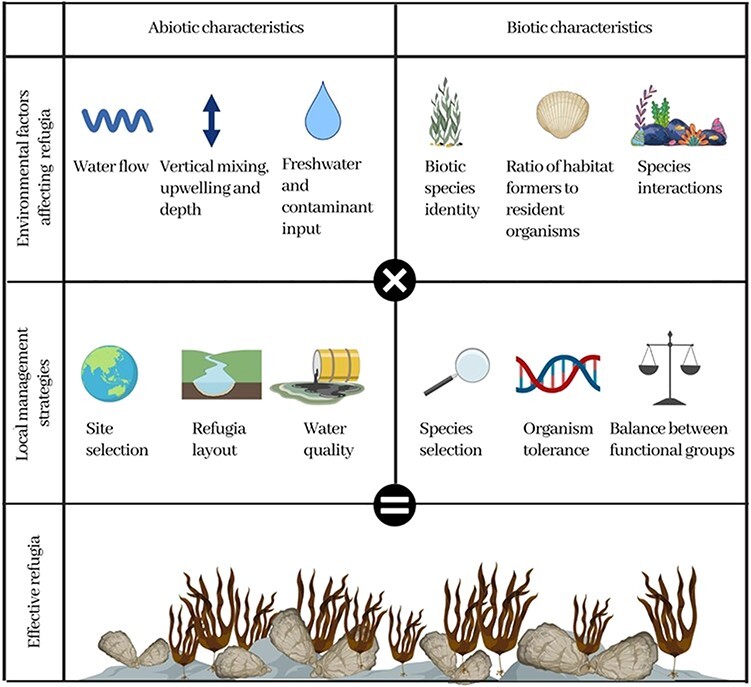
Abiotic and biotic factors can affect refugia success, and these can be targeted via local management strategies that will lead to effective refugia. Specifically, appropriate abiotic characteristics can be enhanced via site selection, refugia layout and water quality management (often nutrient removal). The biotic characteristics can be enhanced via considered selection of species introduced (focusing on those that are suited to current and expected future conditions), maximizing the tolerance of introduced organisms (through processes including species genotype selection, synthetic biology, assisted evolution and microbial management; population-level conservation biology, biodiversity maintenance), and ensuring a balance between functional groups (such as the habitat formers, calcifiers and herbivore predators).

The effects of global ocean acidification will reflect the local adaptation solutions implemented (Gattuso *et al.*, 2015). Promisingly, local management that benefits habitat-forming species may be effective in ensuring the continued acquisition of services from both natural systems and those associated with human activities, such as aquaculture. Such management approaches are increasingly recognized as an important part of restricting the effects of largely unmanageable global change. However, the success of such intervention will depend upon the context in which it is implemented. Consequently, critical aspects of implementation that need to be considered and managed include abiotic and biotic conditions. We propose that where the conditions of the surrounding environment are effectively understood and managed, it will increase the likelihood of effectively ensuring the provision of local refugia for calcifiers in natural systems, and inform approaches to enhance those associated with aquaculture (Fig. 2).

### Abiotic conditions

Modifying seawater chemistry by introducing habitat-forming species will be most feasible where the chosen sites have suitable abiotic traits. That is, success will be greatest in areas where the benefits will not be neutralized by other coastal drivers, such as high flow rates, vertical mixing and upwelling, input of freshwater and contaminants from terrestrial systems, light availability, seasonal effects or other global change parameters. Consequently, it is important that the introduction of habitat-forming species is implemented with recognition of the prevailing hydrodynamic regimes and their implications for the extent of biotic refugia. For example, the footprint of the ocean acidification buffering effect of the seagrass *P. oceanica* (Linnaeus) Delile has been found to extend ~10 m from the meadow boundary (Fernández *et al.*, 2019). While this information will be useful to make decisions about how near habitat-forming species need to be located to the species they are to protect in this location, it is expected that these distances will vary with context. Consequently, of particular importance in the design of an effective cultivation layout will be the recognition of the prevailing abiotic characteristics.

Where the contemporary abiotic conditions are understood, it may be possible to manage them to enhance the capacity of habitat-forming species to act as refugia. In some situations, the abiotic conditions will be modified as a consequence of the introduction of the habitat-forming species. For example, mussel farms, algal canopies and seagrass meadows can reduce current speeds, which affect water residence times, potentially accentuating the buffering effect (Hendriks *et al.*, 2014; Hurd, 2015; Fernández *et al.*, 2019). In other situations, additional local-scale management of coastal drivers could create conditions that enhance the likelihood of success. For example, while elevated nutrients can favour shifts in community structure from canopy-forming macroalgae towards turf-forming algae (Russell *et al.*, 2009; Falkenberg *et al.*, 2013b), it is likely that where nutrients are removed, the probability of such shifts will be reduced (Falkenberg *et al.*, 2013a). Enhancing water quality in an area can even allow macroalgae to return to systems (Hawkins *et al.*, 1999). Moreover, such management could also enhance resilience to other climate stressors. For example, the removal of nutrients can enhance the tolerance of canopy-forming macroalgae to increased temperature (Strain *et al.*, 2015).

### Biotic conditions

Ocean acidification refugia will have the greatest chance of success where care is taken in the enhancement of habitat-forming species. In locations where habitat-forming species, such as kelp, have been present, restricting the causes of kelp decline such as overharvesting by humans can allow for populations of historically present species to return (Buschmann *et al.*, 2014; Frangoudes and Garineaud, 2015). However, there are also many additional global and local causes of kelp decline—such as altered trophic systems (e.g. otter extirpations, overfishing), climate change, marine heatwaves, water quality issues, the spread of invasive species and often some combination of stressors—that result in region-specific responses that make forecasting change and implementing management difficult (Krumhansl *et al.*, 2016). Consequently, management can therefore be logistically and politically complex, or impossible in the case of local actions impacting global stressors. Recovery-centred activities can be supplemented by active introduction aiming to restore lost populations through the transportation of adult or juvenile organisms from a donor site, or the out planting of laboratory-cultured individuals (Layton *et al.*, 2020). In these scenarios, it will be possible to select the species introduced from those that have been historically present. Of particular importance in shaping the suitability of different species are traits associated with their anticipated responses to ocean acidification. In terms of habitats formed by macroalgae and seagrasses this suitability is largely related to their ability to photosynthesize under ocean acidification and, therefore, provide short-term buffering. Briefly, we could expect that macroalgae which benefit from elevated CO_2_ due to enhanced photosynthetic rates would have a greater capacity to modify seawater carbonate chemistry in their surrounding environment. Such traits would likely be beneficial when selecting the organisms to be cultivated under an ocean acidification scenario. Similar approaches are already being used in the management of other global changes. For example, some existing macroalgae restoration programs are selecting algae that is tolerant to heatwaves in order to increase the chances of survival in a warming ocean (Layton *et al.*, 2020).

In terms of the calcifiers, their suitability is likely to be related to their ability to deal with long-term change, and shorter-term fluctuations, in water chemistry conditions. For example, it has been identified that there can be differences in responses to ocean acidification between species, populations and even individuals (Parker *et al.*, 2011; Falkenberg *et al.*, 2019; Fernández *et al.*, 2019, and references therein). Adaptation via the selection of more tolerant genotypes has been identified as a potential source of resilience for some marine species (Parker *et al.*, 2011, 2012; Wright *et al.*, 2014; Foo *et al*., 2014; Scanes *et al.*, 2021). For example, in the sea urchin, *Centrostephanus rodgersii*, the tolerance of early embryos to ocean warming and acidification was found to differ among genotypes, with some genotypes showing no effect of warming and only moderate effects of ocean acidification (Foo *et al.*, 2012). In the Sydney rock oyster, *Saccostrea glomerata*, populations that were selectively bred for faster growth and resistance to disease grew 65% faster and were better able to maintain their extracellular pH under ocean acidification compared to the wild population (Parker *et al.*, 2011, 2012). The response of marine organisms exposed to acute warming was also shown to be dependent on genotypes. Some genotypes of the oyster *S. glomerata* were found to experience up to 50% mortality when exposed to an atmospheric heatwave of 50°C, while there was less than 20% mortality experienced by other genotypes (Scanes *et al.*, 2020b). While these improvements in the response of organisms is encouraging, care still needs to be taken to select organisms that are best suited to the abiotic conditions.

Similarly, the suitability of the species to the general abiotic conditions will be important to ensure success. For example, in terms of the habitat-forming species, depth has been found to be a critical determinant of seagrass restoration success (Aoki *et al.*, 2020). Similarly, macroalgae are also influenced by depth in ways that will influence their capacity to provide refugia. In shallow water (here <3 m depth) brown algae may be appropriate; however, where deeper depths are considered (here, below 4 m depth, up to ~13 m depth), the accessory pigments of red algae would reduce their light limitation and likely make them a better choice (Fernández *et al.*, 2019, and references therein).

Creating habitat refugia will be most successful when the species and populations are chosen to match both the present and future environment. Specifically, habitat formers will have the best chance at providing refugia where individuals are able to tolerate future climatic conditions, and this has been proposed to be possible via human intervention such as (i) genotype selection whereby stress-tolerant or diverse genotypes are selected and undergo assisted reallocation to sites of interest, (ii) synthetic biology to undertake gene editing of the habitat-forming species of interest such that its tolerance to stress is increased, (iii) assisted evolution in which human-assistance accelerates natural processes to build tolerance and (iv) microbial management in which its assisted evolution is prompted or the community is modified via selective removal of particular microbial taxa (further details of each and examples can be found in Bulleri *et al.*, 2018; see also Singh and Reddy, 2014; Coleman and Goold, 2019; Wood *et al.*, 2019; Layton *et al.*, 2020). In addition to enhancing the occurrence of individuals, there are also ways in which human actions could enhance the entire population’s tolerance via management of biotic traits. Approaches include (i) implementation of discipline-specific knowledge from conservation biology to sustain population viability of habitat-forming species via, for example, protection of source populations, restoration or creation of migration corridors or managed relocation; (ii) biodiversity maintenance that would likely benefit resilience and temporal stability, as well as the provision of diverse microhabitats, through active control of competitively dominant species, or seeding of facilitative or subordinate species; (iii) ecoengineering of artificial habitats to supply suitable habitats or new dispersal routes; and (iv) non-native species introduction as alternatives to native habitat formers (although we note that this last suggestion is, in particular, controversial; Bulleri *et al.*, 2018; Eger *et al.*, 2020).

An example of successful restoration can be found in the Hinase region of Japan where local fishermen have replanted seagrass meadows adjacent to oyster aquaculture. The seagrass in this region was lost due to damaging fishing practices; however, by planting seeds over several decades the seagrass meadows have recovered and currently possess a genetic diversity comparable to local undisturbed seagrass meadows (Hori and Sato, 2021). This restoration has also had benefits for oyster production with increased yields and lowered yearly variability (Hori and Sato, 2021). These benefits are likely to be enhanced as ocean acidification strengthens and the seagrasses provide further refugia for oyster aquaculture.

The habitat-forming and refuge species will exist in combination in the established system and, consequently, the optimal proportions between, for example, macroalgae and bivalves, need to be identified and established. Such a ratio would need to ensure sufficient algae to uptake excess CO_2_ during the day and reduce pH, while not releasing too much CO_2_ at night and dropping the pH too low. Similarly, where in balance, there would be sufficient waste excretions from shellfish to favour the growth of macroalgae, but not to the point where other epiphytic algae gain a competitive advantage. Achieving this balance will be particularly important in an aquacultural context, as the greatest biomass and most efficient growth of both culture components needs to be ensured (for example, shellfish aquaculture—mussel farms—and macroalgae; Fernández *et al.*, 2019). Consequently, the physiological responses of both the habitat former and the resident species (e.g. CO_2_ uptake capacity, buffering effects, nutrient uptake and excretion rates) need to be tested when considering a co-culture design (Fernández *et al.*, 2019). Not only will the results of such tests likely be specific to the species being considered, but also to the site being considered.

Finally, this management will occur in the presence of other ecosystem components, which have the potential to modify the balance between the habitat-forming species and resident organisms. Their management will, therefore, also be of consequence. Of particular importance is the management of herbivore populations through controls of predators, which may prime ecosystems for the establishment of habitat-forming macrophytes (Eger *et al.*, 2020). This has been proposed to be possible via both the establishment of harvest limitations and the creation of marine protected areas (MPAs). In terms of harvesting, installing limits on predator harvest has been associated with the return of kelp habitat in Alaska (Estes and Duggins, 1995), California (Caselle *et al.*, 2018), British Columbia (Watson and Estes 2011), Australia (Layton *et al.*, 2020) and New Zealand (Shears and Babcock, 2002). In terms of MPAs, the associated limitation of harvesting of certain marine predators that play a role in determining herbivore population can promote the resilience of macroalgal ecosystems (Ferrari *et al.*, 2018). It is important to note, however, that restriction of these harvesting pressures is often insufficient to allow for the return of predators and, therefore, the habitat-forming species. To resolve this, managers can turn to the translocation and introduction of predators (Bodkin, 2015), which has been successful at restoring and maintaining habitat-forming species (Filbee-Dexter and Scheibling, 2014). Where predators or upper trophic level species are unable to exert sufficient top-down control on herbivore populations, an alternative approach to restrict the occurrence of herbivores is via human intervention and direct removal. In the context of sea urchins and kelp, one innovative solution is the removal of urchins by divers who then sell them (although there may need to be a period of land-based aquaculture to enhance their quality before sale) (Eger *et al.*, 2020, and references therein).

## Conclusions

Habitat-forming organisms influence their surrounding abiotic environment and can modify the stress faced by associated organisms under future environmental conditions. Of particular interest in the context of ocean acidification is the presence of macroalgae canopies and seagrass meadows in providing refugia for resident organisms. Here, we propose that the impact will be dependent on the abiotic and biotic context, which provides local managers the opportunity to create conditions to enhance the likelihood of successful amelioration. Appropriate management would likely include the following: locating activities in sites with suitable abiotic conditions with consideration of the influence of adjacent habitats, managing local abiotic conditions to favour growth (e.g. removing contaminants), introducing appropriate habitat-forming species, managing biota to enhance the tolerance of organisms to climate change, ensuring a balance between habitat formers and resident species, as well as managing the co-occurring biota from different trophic levels. If the introduction and maintenance of habitats is achieved, their presence could reduce the loss of, or even promote an increase in, the value that human societies gain from dependent organisms under ocean acidification scenarios.

## Funding

This work was supported by the University of Sydney-Chinese University of Hong Kong Partnership Collaboration Award in 2019–2020.

## Supplementary material

Supplementary material is available at *Conservation Physiology* online.
